# Chemotherapy alone vs. chemotherapy plus radiotherapy in female adolescent and young adults with Hodgkin’s lymphoma: reproductive health outcomes

**DOI:** 10.1007/s11764-023-01388-z

**Published:** 2023-05-06

**Authors:** Susan Luong, Claire Mockler, Jessica Pudwell, Wenbin Li, Jill Dudebout, Maria P. Velez

**Affiliations:** 1 Department of Obstetrics and Gynecology, Queen’s University, Kingston, Canada; 2 School of Medicine, Queen’s University, Kingston, Canada; 3 ICES Queen’s University, Kingston, Canada; 4 Department of Oncology, Queen’s University, Kingston, Canada

**Keywords:** Chemotherapy, Radiotherapy, Hodgkin’s lymphoma, Infertility, Pregnancy, Menopause

## Abstract

**Purpose:**

To examine the effects of Hodgkin’s lymphoma and its treatment on reproductive health in female adolescent and young adults (AYA).

**Methods:**

We conducted a retrospective, population-based, matched-cohort study of female patients with Hodgkin’s lymphoma diagnosed at 15–39 years of age from 1995 to 2014 in Ontario, Canada. Three female individuals with no history of cancer (unexposed) were matched by birth year and census subdivision to each patient with cancer (exposed). In a subset of the cohort (2005 onwards), the Hodgkin’s lymphoma patients were further classified into two groups for analysis based on treatment exposure: (1) chemotherapy alone or (2) combined chemotherapy and radiation. Reproductive health outcomes were infertility, childbirth, and premature ovarian insufficiency (POI). Relative risks (RR) were calculated using modified Poisson regression adjusted for income quintile, immigration status, and parity.

**Results:**

A total of 1443 exposed and 4329 unexposed individuals formed our cohort. Hodgkin’s lymphoma patients were at an increased risk of infertility (aRR 1.86; 95% CI 1.57 to 2.20) and POI (aRR 2.81; 95% CI 2.16 to 3.65). While the risk of infertility persisted in both treatment groups (chemotherapy alone, combined chemotherapy plus radiotherapy), the increased risk of POI was only statistically significant in the chemotherapy plus radiotherapy group. No differences in childbirth rates were observed, overall or by treatment exposure compared with unexposed individuals.

**Conclusions:**

Female AYA survivors of Hodgkin’s lymphoma face an increased risk of infertility, independent of exposure to chemotherapy alone, or chemotherapy plus radiotherapy. The risk of POI is higher in those requiring radiotherapy vs. chemotherapy alone.

**Implications for cancer survivors:**

These results emphasize the importance of pre-treatment fertility counseling and reproductive health surveillance for AYAs diagnosed with Hodgkin’s lymphoma.

**Supplementary Information:**

The online version contains supplementary material available at 10.1007/s11764-023-01388-z.

## Background

Hodgkin’s lymphoma (HL) is one of the most common cancers diagnosed in adolescents and young adults (AYAs, age 15–39 years) [1]. Modern therapies have dramatically improved the prognosis of HL, which currently has a 5-year survival rate of 86% [2]. Chemotherapy plus radiotherapy or chemotherapy alone is recommended treatment options for patients with HL [3]. Pelvic radiotherapy and some chemotherapy regimens may affect fertility and reduce the reproductive lifespan in patients with HL [4]. Considering that survival rates are increasing and most of the female reproductive years fall within the AYA age range, there needs to be a greater focus on reproductive health outcomes following treatment within this population.

Our group previously assessed the risk of infertility diagnosis and premature ovarian insufficiency (POI, menopause before age 40 years) among female AYA survivors of various cancer types in Ontario at the population-based level [5, 6]. Survivors of HL had a higher risk of infertility diagnosis and POI compared with matched controls [5, 6]. In terms of childbirth, others have reported that childbirth rates are similar among AYA HL survivors compared with patients without cancer [7-9]. The effect of chemotherapy alone vs. chemotherapy plus radiotherapy in survivors of HL needs further investigation. Prior studies included HL survivors treated before 1995 [10, 11], relied on self-reported reproductive outcomes, and did not include a comparison group [10-12]. Our objective, therefore, was to examine the effect of HL and its treatment on reproductive health outcomes in female AYA HL survivors, using a population-based approach in Ontario, Canada from 1995 to 2014.

## Materials and methods

### Study design and population

This population-based cohort study included AYA residents of Ontario diagnosed with HL from January 1995 to December 2014 in the exposed arm. The unexposed arm included three age and geographically matched females with no history of cancer for every one person in the exposed arm. Matching was completed using year of birth and census subdivision, using random selection without replacement. In the exposed arm, index date was defined as date of diagnosis. In the unexposed arm, index date was assigned as the date of diagnosis of their matched exposed participant.

Exclusion criteria for both arms were a history of any cancer prior to index date, a history of a sterilizing procedure (tubal ligation, bilateral oophorectomy and/or hysterectomy) prior to or up to 3 years after index date, a diagnosis of infertility prior to the index date, death within 3 years of index date, missing information on geographical census subdivision, and loss of OHIP eligibility on or within 3 years of the index date (Online Resource, Table A2). Among the exposed arm, additional exclusions were diagnosis of an additional cancer (other than HL) on or within 1 year of the index date and inability to find 3 appropriate unexposed matches. Among the unexposed arm, an additional exclusion of a diagnosis of any cancer on or within 1 year of the index date was applied.

Participants were followed from index date until the end of follow up; defined as date of occurrence of an outcome of interest, date of a new cancer diagnosis, date of death, date of loss of OHIP eligibility, or maximum follow up date — whichever occurred first. For POI, the maximum age of 40 was also used as a censoring date. Any matched pair with an individual over 39 was removed from the POI outcome analysis. The maximum follow up date for the cohort was December 31st, 2019.

### Data sources

Data used for this study included universal coverage administrative health data for Ontario residents available at ICES (http://www.ices.on.ca). ICES is an independent, non-profit research institute whose legal status under Ontario’s health information privacy law allows it to collect and analyze deidentified health care and demographic data for health system evaluation and improvement. The exposed cohort was identified through the Ontario Cancer Registry (OCR). The registry is a comprehensive provincial database that captures at least 98% of incident cancers in Ontario and includes diagnostic and treatment information. A description of all data sources used can be found in the Appendix (Online Resource, Table A1).

### Exposure and outcomes

The exposure of HL malignancy was defined based on morphology codes in the OCR (Online Resource, Table A3). In a subset of the cohort with available data on treatment type (2005 onwards) the exposed arm was further classified based on treatment exposure defined as (1) chemotherapy alone or (2) combined chemotherapy and radiation, as captured in the OCR. In all analyses, the unexposed arm was the referent group. A treatment was considered received if it was found in a record within the OCR within 2 months before and 12 months after cancer diagnosis.

All outcomes were defined based on a window from 1 year after index date until the end of follow up. Reproductive outcomes included infertility, childbirth, and POI (Online Resource, Table A5). Infertility diagnosis was defined as the first occurrence of the International Classification of Disease, Ninth Revision (ICD-9) code 628 (infertility diagnosis) in the Ontario Health Insurance Plan (OHIP) database. Childbirth was defined as any record of a pregnancy event in the MOMBABY with a gestational age at delivery ≥ 20 weeks and an estimated date of conception within the outcome window. POI was defined based on the first occurrence of ICD-9 code 627 (menopause diagnosis) in the OHIP database, as long as the diagnosis occurred before the age of 40 years.

### Patient characteristics

Participant demographic and health history information were obtained from administrative data records of healthcare encounters (Online Resource, Table A4). Information for age, date of birth, and date of death, as well as health insurance eligibility was obtained from the Registered Persons Database (RPDB). Income quintile was assigned using the postal code conversion file (PCCF), with a higher quintile denoting higher income. Rurality of residence was defined using postal code through the rurality index (RIO 0–39 compared to RIO ≥ 40). Participants were classified as immigrant or Canadian born with information from the Immigration Refugees and Citizenship Canada Permanent Resident (IRCC-PR) dataset. Parity prior to index was identified through MOMBABY; a participant was considered parous if a record of a live birth or stillbirth was found, otherwise they were considered nulliparous. History of endometriosis or polycystic ovarian syndrome (PCOS) was defined based on an occurrence of ICD-9 code 617 or 256 in the OHIP database prior to index, respectively.

### Statistical analysis

Sociodemographic characteristics were compared by exposure using standard differences; those with a standardized difference greater than 0.10 were considered a clinically meaningful difference [13]. Modified Poisson regression produced unadjusted relative risks (RR) and 95% confidence intervals for the association between HL and reproductive outcomes further adjusted for income quintile, immigration status, and parity (aRR). Given that age at cancer diagnosis and the evolution of treatment protocols over time may have an impact on the association between HL and reproductive outcomes, two additional analyses were performed, in which the cohort was stratified by age categories (15–29 years vs. 30–39 years), and by study era (1994–2004 vs. 2005–2015). All analyses were completed using SAS software v9.4 (SAS Institute Inc. Cary, NC, USA).

## Results

**Fig. 1:**
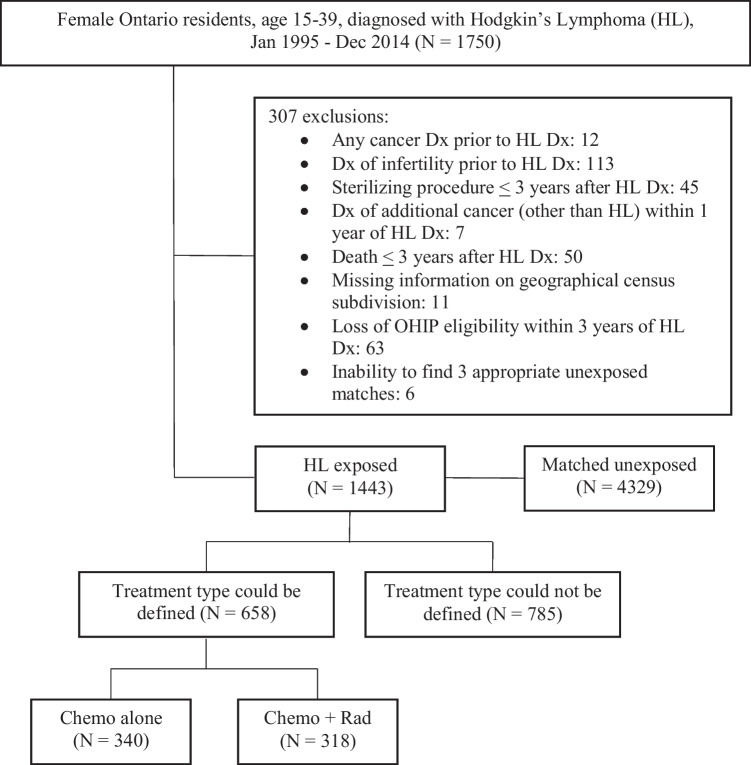
Cohort creation

A total of 1443 exposed females and 4329 matched unexposed females were included in the study (Fig. 1). Average age at index was 25.5 ± 6.6 years and 90.6% of the cohort resided in an urban area (Table 1). Significant differences in immigrant status and income quintile were found with a greater proportion of the exposed cohort being Canadian-born and in the highest income quintile. Other characteristics including age, rurality, parity, and history of PCOS and endometriosis were not different between the arms. Among the exposed cohort for which treatment exposures could be defined (*N* = 686), 49.6% received chemotherapy alone and 46.4% chemotherapy with radiation.

**Table 1:** Characteristics of 5772 female individuals in Ontario, Canada, aged 15–39 years by Hodgkin's lymphoma exposure between January 1995 to December 2014. All data are presented as numbers (%) unless otherwise indicated

Characteristics	Exposed *N* = 1443	Unexposed *N* = 4329	Standardized difference
Age at index date, mean ± SD	25.5 ± 6.6	25.5 ± 6.6	0.003
Income quintile			
1—lowest income	233 (16.0)	907 (21.0)	0.13
2	272 (18.8)	853 (19.7)	0.02
3	293 (20.3)	850 (19.6)	0.02
4	359 (24.9)	897 (20.7)	0.10
5—highest income	286 (20.0)	822 (19.0)	0.02
Rural	136 (9.4)	408 (9.4)	0.0
Immigrant	156 (10.8)	734 (17.0)	0.18
Endometriosis	22 (1.5)	58 (1.3)	0.02
Polycystic ovary syndrome	33 (2.3)	59 (1.4)	0.07
Nulliparous	1118 (77.5)	3207 (74.1)	0.08

Outcomes by exposure status are presented in Table 2. Of the exposed cohort, 274/1443 (19.0%) had infertility diagnosis > 12 months after index date, compared to 499/4329 (11.5%) of those unexposed (standardized difference = 0.21). For those exposed, average age of first infertility consultation was 30.9 ± 5.6 years, compared to 32.6 ± 5.1 years among the unexposed (standardized difference = 0.32). The proportion of births did not differ between the arms. For those exposed, 110/1402 (7.8%) experienced POI, compared to 124/4206 (2.9%) of unexposed (standardized difference = 0.22).

**Table 2:** Hodgkin's lymphoma and associated reproductive outcomes in female adolescents and young adults 15–39 years in Ontario, Canada, 1995–2014

Outcomes	Exposed/no at risk (rate %)	Unexposed/no at risk (rate %)	Standardized difference	Unadjusted RR (95% CI)	Adjusted RR (95% CI)*
Infertility	274/1443 (19.0)	499/4329 (11.5)	0.21	1.94 (1.62 to 2.32)	1.86 (1.57 to 2.20)
Childbirth	509/1443 (35.3)	1607/4329 (37.1)	0.04	0.90 (0.75 to 1.09)	0.90 (0.75 to 1.07)
Premature ovarian insufficiency	110/1402 (7.8)	124/4206 (2.9)	0.22	2.85 (2.19 to 3.71)	2.81 (2.16 to 3.65)

^*^Relative risks were adjusted for income quintile, immigration status, and parity

Unadjusted and adjusted models (aRR) are presented in Tables 2 and 3. Exposure to HL was associated with an increased risk of infertility compared to those who were not exposed (aRR 1.86; 95% CI 1.57 to 2.20). When considering specific treatment exposure, increased risk of infertility was seen for both chemotherapy alone (aRR 2.61; 95% CI 1.75 to 3.89) and chemotherapy with radiation (aRR 1.88; 95% CI 1.27 to 2.79). HL exposed individuals had similar childbirth rates than unexposed individuals (aRR 0.90; 95% CI 0.75 to 1.07), independent of treatment type, chemotherapy alone (aRR 0.78; 95% CI 0.54 to 1.12), or chemotherapy with radiation (aRR 1.05; 95% CI 0.63 to 1.74). Those exposed to HL were at an increased risk of POI (aRR 2.81; 95% CI 2.16 to 3.65) compared to those not exposed. For POI, the association was statistically significant for chemotherapy with radiation (aRR 2.18; 95% CI 1.13 to 4.18), but not for chemotherapy alone (aRR 1.86; 95% CI 0.97 to 3.55).

**Table 3:** Hodgkin's lymphoma stratified by chemotherapy alone vs. chemotherapy plus radiotherapy treatment and associated reproductive outcomes in female individuals aged 15–39 years in Ontario, Canada. This analysis is limited to female individuals with diagnosis of Hodgkin's Lymphoma from 2005 to 2014 in whom treatment type was available vs. unexposed individuals

Outcome	Exposure	Exposed/no at risk (rate %)	Unexposed/no at risk (rate %)	Unadjusted RR (95% CI)	Adjusted RR (95% CI)*
Infertility	Chemotherapy	59 /340 (17.4)	82/1020 (8.0)	2.67 (1.76 to 4.06)	2.61 (1.75 to 3.89)
	Chemotherapy + radiation	30/318 (15.7)	91/954 (9.5)	1.98 (1.27 to 3.08)	1.88 (1.27 to 2.79)
Childbirth	Chemotherapy	92/340 (27.1)	324/1020 (31.8)	0.78 (0.55 to 1.11)	0.78 (0.54 to 1.12)
	Chemotherapy + radiation	95/318 (29.9)	284/954 (29.8)	1.03 (0.61 to 1.74)	1.05 (0.63 to 1.74)
Premature ovarian insufficiency	Chemotherapy	15/328 (4.6)	24/984 (2.4)	1.89 (1.00 to 3.54)	1.86 (0.97 to 3.55)
	Chemotherapy + radiation	18/316 (5.7)	27/948 (2.8)	2.24 (1.15 to 4.38)	2.18 (1.13 to 4.18)

^*^Relative risks were adjusted for income quintile, immigration status, and parity

Stratifying the analysis by age categories (Table 4) did not modify the effect of HL on infertility and childbirth, however AYAs 30–39 had a higher risk of POI (aRR 8.29; 95% 4.29 to 16.03) than AYAs 15–29 years (aRR 2.10; 95% 1.57 to 2.81). Additional analysis by study era (Table 5) did not alter the results.

**Table 4:** Hodgkin's lymphoma and associated reproductive outcomes in female adolescents and young adults stratified by age categories in Ontario, Canada, 1995–2014

Outcomes	Exposed/no at risk (rate %)	Unexposed/no at risk (rate %)	Standardized difference	Unadjusted RR (95% CI)	Adjusted RR (95% CI)*
15–29 years					
Infertility	223/1040 (21.4)	412/3117 (13.2)	0.22	1.86 (1.54 to 2.24)	1.79 (1.49 to 2.14)
Childbirth	431/1040 (41.4)	1,405/3117 (45.1)	0.07	0.87 (0.74 to 1.03)	0.88 (0.75 to 1.04)
Premature ovarian insufficiency	75/1040 (7.2)	109/3117 (3.5)	0.17	2.14 (1.60 to 2.86)	2.10 (1.57 to 2.81)
30–39 years					
Infertility	51/403 (12.7)	87/1212 (7.2)	0.18	2.24 (1.35 to 3.72)	2.26 (1.41 to 3.63)
Childbirth	71/403 (17.6)	196/1212 (16.2)	0.04	0.87 (0.74 to 1.03)	0.88 (0.75 to 1.04)
Premature ovarian insufficiency	35/362 (9.7)	15/1089 (1.4)	0.37	8.57 (4.31 to 17.03)	8.29 (4.29 to 16.03)

^*^Relative risks were adjusted for income quintile, immigration status, and parity

**Table 5:** Hodgkin's lymphoma and associated reproductive outcomes in female adolescents and young adults stratified by study era in Ontario, Canada

Outcomes	Exposed/no at risk (rate %)	Unexposed/no at risk (rate %)	Standardized difference	Unadjusted RR (95% CI)	Adjusted RR (95% CI)*
1994–2004					
Infertility	162/757 (21.4)	318/2271 (14.0)	0.20	1.76 (1.41 to 2.19)	1.69 (1.35 to 2.12)
Childbirth	306/757 (40.4)	968/2271 (42.6)	0.05	0.91 (0.70 to 1.19)	0.92 (0.75 to 1.11)
Premature ovarian insufficiency	74/732 (10.1)	72/2196 (3.3)	0.28	3.28 (2.37 to 4.54)	3.18 (2.31 to 4.39)
2005–2015					
Infertility	112/686 (16.3)	181/2058 (8.8)	0.23	2.28 (1.68 to 3.08)	2.20 (1.67 to 2.90)
Childbirth	196/686 (28.6)	633/2058 (30.8)	0.05	0.89 (0.68 to 1.16)	0.88 (0.67 to 1.16)
Premature ovarian insufficiency	36/670 (5.4)	52/2010 (2.6)	0.14	2.22 (1.42 to 3.48)	2.21 (1.41 to 3.46)

^*^Relative risks were adjusted for income quintile, immigration status, and parity

## Discussion

Overall, we found that HL survivors were at increased risk of infertility and POI compared to our matched cohort with no history of cancer. We further classified our HL group according to treatment type to examine that impact of chemotherapy alone compared to chemotherapy with radiation. Both treatment types increased the risk of infertility and POI. No difference in childbirth rates were observed overall or by treatment exposure in our study population. While era of HL diagnosis did not modify the effect estimates on three outcomes studied, the risk of POI was four times higher in AYAs 30–39 years than in those 15–29 years.

Our finding that HL survivors are at increased risk of infertility is consistent with other groups who have reported high rates of infertility and issues with ovarian function within this population. One group assessed self-reported fertility status among 36 female HL survivors of reproductive age and found that 22% perceived themselves as infertile [14]. Another study reported that 32% of women experienced amenorrhea after treatment for HL [11]. Our study expands on these findings by quantifying the risk compared to an unexposed cohort and using diagnostic codes rather than self-reports. Of note, the average age of infertility diagnosis was 2 years earlier in HL survivors than unexposed individuals, which could reflect increased counseling by the health care team about the reproductive impact of cancer therapies as recommended by current practice guidelines [15]. In terms of treatment type, although the difference in effect size for infertility with chemotherapy alone vs. chemotherapy plus radiotherapy is non-significant (given that the 95% CI overlap), a lower aRR for the combined therapy group could reflect the lower cumulative doses of chemotherapy that radiotherapy allows. In fact, there is a difference in pediatric vs. adult approaches to treatment and younger AYA may be treated with either approach with varying exposures [16]. Further studies are needed to evaluate this hypothesis.

While our group found that 7.8% of those exposed to HL later experienced POI, other studies report higher rates of POI among HL survivors. A study conducted in the Netherlands found that 20% of HL survivors developed POI [17] and a Norwegian study reported a rate of 37% [18]. The higher rates of POI reported in these studies may be explained by the use of questionnaires for data collection, rather than diagnostic codes, which would capture individuals who had yet to seek medical advice. In addition, differences among studies may be influenced by different treatment protocols with different gonadotoxic potential.

Alkylating agents, which are used in first-line HL treatment regimens, have previously been shown to increase the risk of infertility, with the risk further increased with the cumulative dose [19]. Toxicity to ovarian function can occur through impairment of follicular maturation or depletion of primordial follicles [20]. Alkylating agents cause follicular and oocyte depletion, by producing covalent bonds between DNA strands, rendering cleavage impossible during replication. This depletion results in amenorrhea and ovarian failure, in addition to damaging the steroid producing granulosa cells [20]. Negative feedback on the HPO axis results in an increased FSH secretion, which in turn triggers a further recruitment of pre-antral follicles exposed to the gonadotoxic effects of chemotherapy [21]. The cumulative dose of alkylating agents and the risk associated with salvage therapy, including conditioning and autologous or allogeneic transplantation, are known to confer high rates of infertility [22]. Further, the degree of toxicity to ovarian function has been shown to vary greatly depending on the specific chemotherapy regimen used. Doxorubicin, bleomycin, vinblastine, and dacarbazine (ABVD) is the chemotherapy regimen of choice in North America [3]. In reserved circumstances escalated bleomycin, etoposide, doxorubicin, cyclophosphamide, vincristine, procarbazine, and prednisone (escalated BEACOPP) is indicated. A couple of recent studies have shown better ovarian function following ABVD (particularly in patients < 35 years of age) compared to BEACOPP [23, 24].

We found no difference in childbirth rates between HL survivors and the matched cohort, which is consistent with a number of previous studies [7, 8, 25]. One prospective, longitudinal study found that the frequency of parenthood did not differ between a cohort of female HL survivors and the German population [8]. Hodgson et al. similarly found little difference in pregnancy rates and median time to pregnancy between female HL survivors and friend or sibling controls [25]. A Danish population–based study reported that Assisted Reproductive Technology (ART) was used more frequently among HL survivors, which provides a possible reason for why similar childbirth rates may be seen between HL survivors and controls despite HL survivors having higher infertility rates [9]. Another group in Ontario conducted a population-based study examining childbirth rates in recurrent-free female survivors of non-gynecological malignancies [7]. Similar childbirth rates were observed overall between HL survivors and matched controls. However, when stratified based on childbirth prior to diagnosis, those who had children pre-diagnosis were less likely to have a child post-diagnosis. Differing attitudes around future parenthood may play a role in this, as young childless cancer survivors may be more likely to want future children and less likely to worry about the consequences of cancer treatment on the health of their future children compared to those who were parents at the time of diagnosis [26]. Studies that further explore the use and success of ART among HL survivors within these populations, or identify social and psychological factors influencing pregnancy following HL treatment, could help explain the discordance between existing studies.

One major strength of this study is the large sample size made possible through the use of province wide electronic health care administrative databases and data spanning two decades of the Ontario Cancer Registry. Another strength is the population-based matched cohort design which allowed us to compare the risk of reproductive health outcomes to non-HL controls. Limitations include the inability to carry out an analysis on specific chemotherapy regimens to further characterize different treatment effects given the absence of this information in the linked datasets. However, in North America, ABVD is the most common regimen for patients with HL [3]. Regarding radiation treatment, in a subset of the cohort (2005 onwards), we were able to identify exposure to radiation therapy as a binary outcome (yes/no); however, the dose and body region exposed was not available. As a proxy of evolution in chemotherapy regimens and radiation dosing/fields we stratified the analysis by study era (1994–2004 and 2015–2014), which resulted in similar effect estimates. Another limitation is the absence of information about relapse and the need for auto or allogenic stem cell transplantation, which will impact infertility and POI rates. In addition, the use of ART [25], which might impact childbirth rates was not available. Also, although our results are adjusted for income quintile and immigration status, other sociodemographic and clinical factors that can impact reproductive outcomes were not available in our datasets (e.g., smoking, drug abuse, body mass index). We also acknowledge that our study only involves HL survivors who sought care for infertility or POI and did not include those who did not seek medical attention.

Despite the limitations, our findings demonstrate that AYAs with HL have increased risk of infertility and POI, independent of treatment modality. With excellent survival rates as a result of improved treatment options for HL, it is necessary that late toxicities of treatment are discussed with patients. Our study highlights the need for appropriate fertility counseling with those of reproductive age at the time diagnosis [15, 27].

### Supplementary Information

Below is the link to the electronic supplementary material.

Supplementary file1 (PDF 180 KB)

## Data Availability

The data set from this study is held securely in coded form at ICES. Although data-sharing agreements prohibit ICES from making the data set publicly available, access may be granted to those who meet prespecified criteria for confidential access, available at www.ices.on.ca/DAS. The full data set creation plan and underlying analytic code are available from the authors upon request, understanding that the computer programs may rely upon coding templates or macros.
