# Supplementary material for: Chemotherapy alone vs. chemotherapy plus radiotherapy in female adolescent and young adults with Hodgkin’s lymphoma: reproductive health outcomes
Source: J Cancer Surviv. 2023 May 6;18(4):1434–41. doi: 10.1007/s11764-023-01388-z (PMC11324694; doi:10.1007/s11764-023-01388-z)
Supplement: Supplementary file 1 — Supplementary file1 (PDF 180 KB) [file 11764_2023_1388_MOESM1_ESM.pdf]

Online Resource: Appendix

Chemotherapy alone vs. Chemotherapy plus radiotherapy in female adolescent and young adults with Hodgkin's Lymphoma: Reproductive health outcomes

Journal of Cancer Survivorship

Susan Luong[1], Claire Mockler[2], Jessica Pudwell[1], Wenbin Li[3], Jill Dudebout[4], Maria P. Velez[1,3]

[1]Department of Obstetrics and Gynecology, Queen's University

[2]School of Medicine, Queen's University

[3]ICES Queen's University

[4]Department of Oncology, Queen's University

Corresponding author:

Maria P. Velez

Email address: maria.velez@queensu.ca

Table A1. Data sources accessed at ICES

**Dataset**	**Description**
**Health Services**	
Canadian Institute for Health Information Discharge Abstract Database (CIHI DAD)	The DAD is compiled by the Canadian Institute for Health Information and contains administrative, clinical (diagnoses and procedures/interventions), demographic, and administrative information for all admissions to acute care hospitals, rehab, chronic, and day surgery institutions in Ontario. At ICES, consecutive DAD records are linked together to form 'episodes of care' among the hospitals to which patients have been transferred after their initial admission.
National Ambulatory Care Reporting System (NACRS)	The NACRS is compiled by the Canadian Institute for Health Information and contains administrative, clinical (diagnoses and procedures), demographic, and administrative information for all patient visits made to hospital- and community-based ambulatory care centres (emergency departments, day surgery units, hemodialysis units, and cancer care clinics). At ICES, NACRS records are linked with other data sources (DAD, OMHRS) to identify transitions to other care settings, such as inpatient acute care or psychiatric care.
Ontario Health Insurance Plan Claims Database (OHIP)	The OHIP claims database contains information on inpatient and outpatient services provided to Ontario residents eligible for the province's publicly funded health insurance system by fee-for-service health care practitioners (primarily physicians) and "shadow billings" for those paid through non-fee-for-service payment plans. The main data elements include patient and physician identifiers (encrypted), code for service provided, date of service, associated diagnosis, and fee paid.
Same-Day Surgery (CIHI SDS)	The SDS is compiled by the Canadian Institute for Health Information and contains administrative, clinical (diagnoses and procedures), demographic, and administrative information for all patient visits made to day surgery institutions in Ontario. The main data elements include patient demographics, clinical data (diagnoses, procedures, physician), administrative data (institution/hospital number etc.), financial data, service-specific data elements for day surgery and emergency.
**Population and Demographics**	
Registered Persons Database (RPDB)	The RPDB provides basic demographic information (age, sex, location of residence, date of birth, and date of death for deceased individuals) for those issued an Ontario health insurance number. The RPDB also indicates the time periods for which an individual was eligible to receive publicly funded health insurance benefits and the best known postal code for each registrant on July 1st of each year.

Postal Code Conversion File (PCCF)	The PCCF database will link to postal codes within a given cohort and determine other census geographic identifiers such as, dissemination/enumeration area, census division, longitude/latitude, urban/rural flag and neighbourhood income quintile.
Patient Contact and Eligibility Yearly Files (CONTACT)	Once a person becomes eligible for OHIP an initial record is created with a start date and an infinite end date. When eligibility ends a second record is created with the same start date but a finite end date.
Immigration Refugees and Citizenship Canada Permanent Resident (IRCC-PR) Database	The Ontario portion of the IRCC-PR Database includes immigration application records for people who initially applied to land in Ontario since 1985. The dataset contains permanent residents' demographic information such as country of citizenship, level of education, mother tongue, and landing date. New immigrants who are currently residing in Ontario but originally landed in another province are not captured in this dataset.
**ICES Derived Cohorts**	
Linked Delivering Mothers and Newborns (MOMBABY)	The ICES MOMBABY Database is an ICES-derived cohort that links the DAD inpatient admission records of delivering mothers and their newborns. From 2002 onward, this linkage is performed deterministically using a maternal-newborn chart matching number. Prior to 2002, mothers were linked to their children by matching on the institutions they were admitted, their postal codes, and their admission/discharge dates.
**ICES Acquired Cohorts**	
Ontario Cancer Registry (OCR)	The OCR is a comprehensive provincial database that captures at least 98% of incident cancers in Ontario and includes diagnostic and treatment information.
Cancer Activity Level Reporting (ALR)	The data elements constitute patient level activity within the cancer system focused on radiation and systemic therapy services and outpatient oncology clinic visits. The dataset contains clinical, patient level data.

Table A2. Exclusion criteria – sterilizing procedures

**Procedure**	**Applicable codes**	**Database**
Tubal Ligation	[CCP] 781, 7810, 782, 7820, 7821, 7822<br><br>[CCI] 1RF51, 1RF59, 1RF89	DAD/SDS/NACRS
Bilateral Oophorectomy	[CCP] A single code of 774, 7741, 775, 7751, 7742, 7752<br>OR Two separate surgeries with code of 772, 773<br><br>[CCI] A single code of 1RB89.DA/LA/RA or 1RD89.DA/LA/RA with attribute code 'B' for bilateral<br>Two separate surgeries with code of 1RB89.DA/LA/RA or 1RD89.DA/LA/RA (attribute code may be 'L' for left, 'R' for right or unknown/blank)	DAD/SDS/NACRS

Hysterectomy	[CCP] 802, 803, 804, 805, 806<br><br>[CCI] 1RM87LA-GX with extent attribute SU<br>1RM87DA-GX with extent attribute SU<br>1RM89AA/CA/DA/LA<br>1RM91AA/CA/DA/LA	DAD/SDS/NACRS

Table A3. Codes and descriptions for group classifications

Group	Applicable codes	Database
HL malignancy	96503, 96513, 96523, 96533, 96543, 96553, 96593, 96613, 96623, 96633, 96643, 96653, 96673	OCR
Chemotherapy	Billing codes G381, G281, G339, G345, G359, G390	OHIP
Chemotherapy and radiation	Billing codes G381, G281, G339, G345, G359, G390<br>+<br>NHPIP codes 500, 501, 503, 510, 511, 512, 519, 520, 521, 522, 523, 524, 525, 526, 527, 528, 529, 530, 531, 532, 533, 534, 535, 536, 537, 538, 539, 540, 541, 542, 543, 544, 548, 549, 561, 563, 565, 566, 568, 570, 571, 572, 573, 574, 575, 576, 577, 581, 582, 590, 591, 592, 593, 594, 595, 596, 597, 598, 599	OHIP/ALR

Table A4. Baseline Characteristics

Variable	Applicable codes	Database
Age group at Cancer Diagnosis	Based on date of birth<br>1) 15-19<br>2) 20-24<br>3) 25-29<br>4) 30-34<br>5) 35-39	RPDB
Income quintile	1) Lowest quintile<br>2) Second quintile<br>3) Third quintile<br>4) Fourth quintile<br>5) Highest quintile<br>If unknown, coded as (1)	PCCF and CENSUS
Rurality Index	1) Urban (RIO 0-39)<br>2) Rural (RIO $\geq$ 40)<br>If unknown, coded as (1)	PCCF and CENSUS
Immigration status	1) Immigrant, Refugee or Other<br>2) Canadian born	IRCC-PR
Parity	1) Parous – any birth (live or stillbirth) prior to the index date<br>2) Nulliparous – no evidence of live or stillbirth prior to index date	MOMBABY
History of Endometriosis	Diagnostic code 617	OHIP
History of PCOS	Diagnostic code 256	OHIP

Table A5. Outcome Classifications

Outcome	Applicable codes	Database
Infertility	Diagnostic code 628	OHIP

Childbirth	Any record of a pregnancy event with a gestational age at delivery ≥20 weeks and an estimated date of conception from 1 year after index date until end of follow up	MOMBABY
Premature Ovarian Insufficiency (POI)	Diagnostic code 627 before the age of 40	OHIP
